# In Vivo Luciferin–Luciferase Reaction in Micro-Mini Pigs Using Xenogeneic Rat Bone Marrow Transplantation

**DOI:** 10.3390/ijms25168609

**Published:** 2024-08-07

**Authors:** Tomoyuki Abe, Kazuhiro Endo, Yutaka Hanazono, Eiji Kobayashi

**Affiliations:** 1 Center for Development of Advanced Medical Technology, Jichi Medical University, Tochigi 329-0498, Japan; 2 Division of Regenerative Medicine, Center for Molecular Medicine, Jichi Medical University, Tochigi 329-0498, Japan; 3 Department of Kidney Regenerative Medicine, The Jikei University School of Medicine, Tokyo 105-8461, Japan

**Keywords:** *luciferase*-transgenic rat, bioluminescent imaging, cell transplantation, bone marrow cells, pigs

## Abstract

Luminescent technology based on the luciferin–luciferase reaction has been extensively employed across various disciplines as a quantitative imaging modality. Owing to its non-invasive imaging capacity, it has evolved as a valuable in vivo bioimaging tool, particularly in small animal models in fields such as gene and cell therapies. We have previously successfully generated rats with a systemic expression of the *luciferase* gene at the *Rosa26* locus. In this study, we transplanted bone marrow from these rats into micro-mini pigs and used in vivo imaging to non-invasively analyze the dynamics of the transplanted cells. In addition, we established that the rat-to-pig transplantation system is a discordant system, similar to the pig-to-human transplantation system. Thus, rat-to-pig transplantation may provide a clinically appropriate large animal model for pig-to-human xenotransplantation.

## 1. Introduction

Imaging-technology-based methods are widely used to observe the structures and functions of different organs within the body. These methods facilitate non-invasive and temporal observations and make a key contribution to translational research. Imaging tools for large animals such as pigs include modalities based on a range of different principles, including computed X-ray tomography (CT), magnetic resonance imaging (MRI), positron emission tomography (PET), and ultrasonography. These technologies are characterized by distinct properties and can be used for multiple purposes. Recent important advances in imaging technology have not only improved the utility of conventional modalities but have also led to the development of new approaches, such as photoacoustic imaging [1] and functional near-infrared spectroscopy [2,3], which enable the acquisition of qualitative and quantitative information from live organisms.

Among imaging technologies, luciferin–luciferase reaction-based luminescence has been used in multiple research fields as a quantitative imaging technique [4,5]. Owing to its non-invasive capacity, this technique is widely used for the in vivo bioimaging of small animals in the fields of gene and cell therapy. Although to date, luciferase bioimaging for large animals, including pigs, has generally been performed using ex vivo excised materials such as brains and hearts [6,7,8], recently, an in vivo study using this imaging technology was performed on live micro-mini pigs [9,10]. In this study, Watano et al. [9] evaluated the delivery of an adeno-associated virus (AAV)-based gene therapy vector for targeting the liver. Among the several AAV vector serotypes assessed, AAV8 was found to be highly directed toward hepatocytes, and the systematic injection of an AAV8 vector carrying the *luciferase* gene resulted in localized gene delivery to the pig liver 7 days after injection [9]. In further studies, Kremen et al. [10] examined the efficacy of cell transplantation therapy for ligament injury in pigs, which involved the transplantation of human mesenchymal cells transduced with a lentiviral vector carrying a *luciferase* gene into the knee joint of a pig with an anterior cruciate ligament transection. Luminescent imaging of the knee joints of the recipient pig, performed 2 weeks after transplantation, revealed that the transplanted human mesenchymal cells had survived in vivo [10].

The *luciferase*-transgenic rats developed by our research team comprise, to the best of our knowledge, the first rat line capable of ubiquitously expressing the *luciferase* gene using the *Rosa26* promoter and have contributed significantly to the advancement of research in the field of transplantation and regenerative medicine [11]. The important contributions of these *luciferase*-transgenic rats include the following: (i) the visualization of injected mesenchymal stem cells distributed to the knee cartilage [12], (ii) visualization of transplanted cardiac muscle sheets and myocardial spheres for the construction of cardiovascular tissues [13,14], and (iii) the in vitro and in vivo development of 3D printer-generated liver buds and organoids grown from small intestinal epithelial cells [15,16]. In this study, we show our findings regarding the dynamics of xenogeneic cells in micro-mini pigs transplanted from *luciferase*-transgenic rats.

Recently, the transplantation of pig hearts and kidneys into humans has attracted increasing attention [17,18]. Given that humans have naturally occurring antibodies against pig antigens, the xenotransplantation of pig organs into human subjects leads to hyperacute rejection minutes after transplantation, and thus xenotransplantation of pig organs into human is categorized as discordant [19]. In contrast, the xenotransplantation of human cells into pigs, as described by Kremen et al. [10], is categorized as concordant and does not cause hyperacute rejection. However, whether xenotransplantation from rats to pigs is discordant has yet to be established. Accordingly, in this study, we aimed to address this question based on the in vivo tracking of rat cells in micro-mini pigs.

## 2. Results

**Figure 1:**
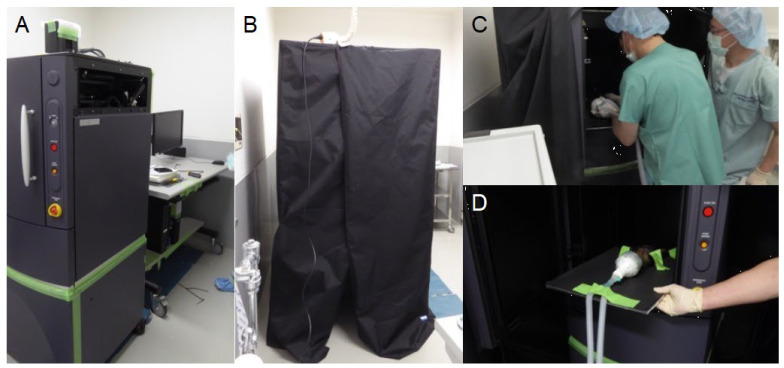
IVIS devices and in vivo imaging of a transplanted micro-mini pig. An overview of the IVIS imaging devices before (**A**) and after (**B**) setting the light-shielding curtains. A pig after the transplantation of bone marrow cells from a *luciferase*-transgenic rat was laid in this apparatus under inhalation anesthesia (**C**) and, subsequently, imaging was performed (**D**).

Bone marrow cells collected from *luciferase*-transgenic rats were transplanted into two micro-mini pigs under general anesthesia. Each pig was transplanted with 1.1 × 10^9^ cells. Following transplantation, an IVIS device was used to analyze the dynamics of the transplanted rat cells in these pigs, as shown in Figure 1.

**Figure 2:**
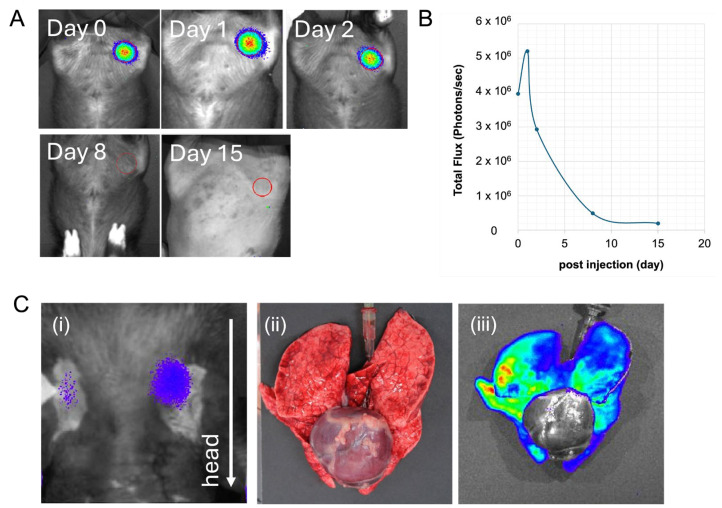
Detection of *luciferase*-transgenic rat cells after transplantation. (**A**) Imaging was performed on days 0, 1, 2, 8, and 15 after intra-bone marrow transplantation. (**B**) The temporal pattern of luminescence intensity (total flux) at the region of interest indicated by red circles in A. Strong luminescence was detected until day 2 after intra-bone marrow transplantation, although declined thereafter and was almost undetectable at 15 days post-transplantation. (**C**) Accumulation of intravenously transplanted rat cells in micro-mini pig lungs. (**i**) Strong luminescence is observed in the chest of a pig. A white arrow indicates the direction of the head. (**ii**) Images of the lungs and heart at autopsy. The pig lungs are colored dark purple. (**iii**) IVIS imaging of the lungs and heart. *Luciferase*-transgenic rat cells are shown to be distributed throughout the lungs.

The micro-mini pig that received rat cells directly into the bone marrow showed no abnormal signs, such as decreased blood pressure and an abnormal heartbeat suggesting hyperacute rejection, and was placed in the IVIS device immediately after transplantation. Strong luminescence was detected in the transplanted femur (Figure 2A). After the pig awakened, luminescence was temporally examined. Although detected at the site of transplantation after 1, 2, and 8 days, the luminescence had become almost undetectable by day 15 post-transplantation (Figure 2A). However, no luminescence was detected in the lungs. Quantitative analysis revealed that the luminescence in the pig was retained for approximately 1 week, although the intensity declined with time (Figure 2B).

In contrast, the second micro-mini pig that received rat cells via a central venous catheter suffered a sudden reduction in blood pressure and an irregular heartbeat, and died of respiratory failure within 20 min after transplantation. Imaging using the IVIS device revealed an accumulation of transplanted cells in the lungs, indicating a pulmonary embolism (Figure 2C).

**Figure 3:**
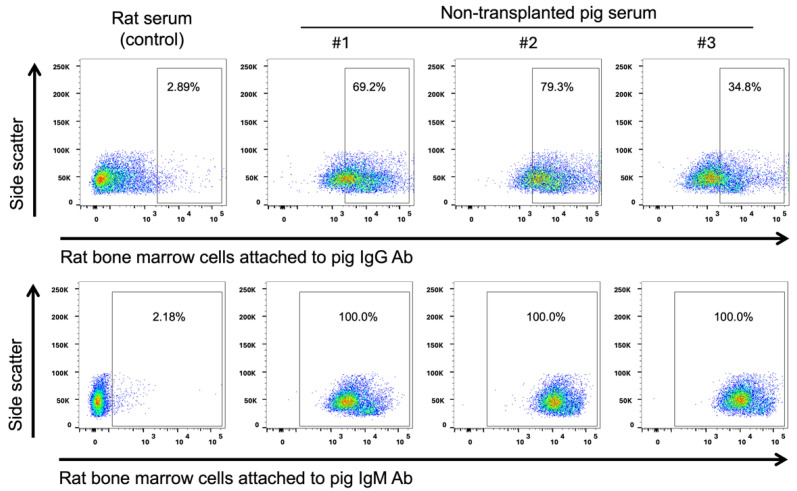
Detection of anti-rat cells antibodies in pigs. Pig IgG (**upper**) and pig IgM (**lower**) against rat cells were determined by flow cytometry. Serum was collected from rats (control) and three non-transplanted micro-mini pigs. Rat bone marrow cells treated with these serums were stained with anti-pig IgG and anti-pig IgM antibodies and analyzed using flow cytometry. From the blood of three non-transplanted micro-mini pigs, pig IgG and IgM against rat bone marrow cells were detected at considerably higher percentages than in the control.

If rat-to-pig transplantation is discordant, xeno-reactive antibodies against rat cells should naturally exist in pigs. Therefore, we examined the presence of immunoglobulin (Ig) G and IgM against rat bone marrow cells in non-transplanted micro-mini pig serum. Pig IgG and IgM against rat bone marrow cells were detected in three non-transplanted micro-mini pig serum at considerable levels compared to control allogeneic (rat) serum (Figure 3).

## 3. Discussion

Recent advances in luminescence imaging have facilitated the detection of luciferin–luciferase luminescence in a range of models, including live pigs. In the present study, luminescence imaging was successfully used to non-invasively observe the in vivo behavior of transplanted xenogeneic rat cells in micro-mini pigs over a period of 15 days. In the case of intravenous transplantation, *luciferase*-transgenic rat cells rapidly accumulated in the pulmonary circulation, resulting in pulmonary embolism, a typical manifestation of hyperacute rejection [19,20,21], thereby indicating that rat-to-pig xenotransplantation is likely to be discordant. Similarly, the transplantation of porcine hearts and kidneys into humans that has recently attracted public attention is a discordant process [17,18]. The present rat-to-pig setting may thus serve as a clinically relevant model for pig-to-human xenotransplantation. Pigs used for organ transplantation undergo multiple genetic modifications to prevent hyperacute rejection [22,23,24,25,26,27]. Although clinical data regarding the extent to which such hyperacute rejection can be prevented by using genetically modified pigs is accumulating, sufficient regulation of xeno-reactive antibodies has not yet been achieved [22,28], indicating that clinically appropriate animal models remain useful. The generation of *luciferase*-transgenic rats with these genetic modifications should facilitate clinically relevant discordant xenotransplantation trials using pigs as recipients.

We found that when xenogeneic rat cells were directly transplanted into the micro-mini pig bone marrow, the transplanted cells engrafted in the bone marrow for several days in the absence of any apparent hyperacute rejection (Figure 2). Bone marrow may be an immune-privileged site, given that it contains high levels of CD4^+^CD25^+^FoxP3^+^ regulatory T cells [29,30]. Other known immune-privileged sites include the eyes, brain, and uterus during pregnancy [31]. However, on the basis of our observation that the number of rat cells engrafted into the pig bone marrow gradually declines with time (Figure 2), it appears that additional strategies will be necessary to achieve stable and longer engraftment time periods.

Luminescence detection devices such as IVIS are typically used for small-spatial-scale experiments, such as small animal experiments, ex vivo experiments with small excised organs, and in vitro experiments with cultured cells [5,32]. However, the findings of the present study illustrate that these devices can also be used in vivo in large animal studies, such as those using micro-mini pigs. These findings thus provide evidence to indicate that luciferase is a powerful tool for obtaining large animal bioimaging data that cannot be obtained with other imaging devices such as CT.

To date, the materials used for luminescence imaging have been based on transgenic firefly-derived genes or the corresponding mutants, as opposed to experimental animal-derived genes [33,34,35], and these luminescent proteins itself induces immune responses in experimental animals. Unless recipient animals are immunodeficient, allogeneic marker genes should be designed and used for long-term in vivo studies. This problem currently hinders the translation of this technology into clinical practice, thereby highlighting the necessity to overcome these limitations for clinical applications.

## 4. Materials and Methods

Bone marrow cells were harvested from *luciferase*-transgenic rats and used for transplantation. The *luciferase*-transgenic rats (6–12 weeks old) were euthanized and their tibias and femurs were obtained. Having cut off the bone ends, the bone marrow was flushed out, and mononuclear cells were isolated using Ficoll (Cytiva, Tokyo, Japan) density centrifugation. The cells were counted, suspended in phosphate-buffered saline (PBS) containing 1% inactivated autologous (recipient micro-mini pig) serum, and used for transplantation.

Treatment and transplantation procedures were performed on micro-mini pigs bred and maintained at Fuji Micra Co., Ltd. (Shizuoka, Japan). All surgical procedures were performed under general anesthesia. The pigs were housed in cages under temperature- and light-controlled conditions (12 h light/dark cycle) and given free access to food and water. From 12 h prior to surgery, the pigs were fasted but had free access to water. The micro-mini pigs were anesthetized with midazolam (Dormicum, Astellas Pharma Inc., Tokyo, Japan) and medetomidine (Domitor, Orion, Turku, Finland), followed by sevoflurane inhalation (Pfizer Japan Inc., Tokyo, Japan), and their vital signs were monitored in accordance with the stipulated guidelines. The ear vein was cannulated and buprenorphine hydrochloride (Lepetan, Otsuka Pharmaceutical Co., Ltd., Tokyo, Japan) was administered for pain relief. A 14-gauge central venous catheter was inserted into the right external jugular vein. To minimize immune rejection, tacrolimus (0.5 mg/kg), mycophenolate mofetil (60 mg/kg), and prednisolone (20 mg/body) were administered orally for five consecutive days prior to transplantation. As a conditioning treatment for transplantation, 6 mg/kg busulfan (Wako Pure Chemical Industries, Ltd., Osaka, Japan) was administered intravenously via a central venous catheter placed in the jugular vein 2 days prior to transplantation, as described by Abe et al. [36]. For intravenous transplantation, *luciferase*-transgenic rat-derived bone marrow cells suspended in PBS containing 1% autologous serum were injected via a central venous catheter. For intra-bone-marrow transplantation, the right femoral bone canal was exposed, and a single puncture was made using an 18-gauge needle to avoid penetrating the bone. After checking for the backflow of bone marrow blood, we injected *luciferase*-transgenic rat-derived bone marrow cells suspended in PBS containing 1% inactivated autologous serum, followed by injection of PBS containing 1% inactivated autologous serum without cells. After removing the needle, the bleeding was stemmed by applying pressure for several minutes. The surgical site was then sutured, and the animal was allowed to awaken spontaneously whilst being kept warm.

Transplanted *luciferase*-transgenic rat cells were analyzed using an IVIS Spectrum CT imaging system (Caliper Life Sciences, Hopkinton, MA, USA) in a room fitted with a light-shielding curtain, with an additional curtain for further shielding (Figure 1A,B). To evaluate luciferase expression in the pigs, a 15 mg/mL D-luciferin solution (OZ Biosciences, San Diego, CA, USA) was injected into the unused cervical vein immediately prior to placing the animal in the IVIS device for imaging (Figure 1C,D).

To detect anti-rat antibodies in pigs, the presence of pig IgG and IgM against rat bone marrow cells was examined using flow cytometry. Rat bone marrow cells were incubated with 10 mL of non-transplanted pig serum diluted 10-fold in PBS(-) for 1 h, at 4 °C. They were then stained with PE-conjugated goat anti-pig IgG antibody (SouthernBiotech, Birmingham, AL, USA) or FITC-conjugated goat anti-pig IgM antibody (Bethyl Laboratories, Montgomery, TX, USA) followed by analysis using a FACS LSRFortessa flow cytometer (Becton Dickinson, Mountain View, CA, USA).

## 5. Conclusions

In this study, we showed that luciferase bioimaging enables the non-invasive and temporal analysis of transplanted cells in micro-mini pigs. We also established that the rat-to-pig xenotransplantation setting is probably discordant, similar to that observed in the pig-to-human clinical setting. The rat-to-pig setting may thus provide a clinically relevant large animal model for pig-to-human xenotransplantation. However, although the rat-to-pig xenotransplantation setting was shown to be discordant, we demonstrated that rat bone marrow cells could be directly transplanted into pig bone marrow in the absence of a hyperimmune rejection.

## Data Availability

Data is contained within the article.
